# Impacts of ectomycorrhizal forest change on nutrient cycling, forest resilience, and ecosystem services

**DOI:** 10.1093/ismejo/wrag103

**Published:** 2026-04-25

**Authors:** Martin I Bidartondo, Sietse van der Linde, Carrie Andrew, Gaby Deckmyn, Guillaume Delhaye, Omar Flores, Jill Kowal, Thomas W Kuyper, Laura M Suz

**Affiliations:** Life Sciences, Imperial College London, London, SW72AZ, United Kingdom; Ecosystem Stewardship, Royal Botanic Gardens, Kew, Richmond, TW93DS, United Kingdom; Netherlands Institute for Vectors, Invasive Plants and Plant Health (NIVIP), National Plant Protection Organisation (NPPO), Netherlands Food and Consumer Product Safety Authority (NVWA), Wageningen, 6706 EA, The Netherlands; Ecosystem Stewardship, Royal Botanic Gardens, Kew, Richmond, TW93DS, United Kingdom; Plant and Vegetation Ecology, University of Antwerpen, Antwerpen, 2000, Belgium; Ecosystem Stewardship, Royal Botanic Gardens, Kew, Richmond, TW93DS, United Kingdom; Earth Sciences, Vrije Universiteit Amsterdam, Amsterdam, 1081 HV, The Netherlands; Ecosystem Stewardship, Royal Botanic Gardens, Kew, Richmond, TW93DS, United Kingdom; Soil Biology, Wageningen University and Research, Wageningen, 6700 AA, The Netherlands; Ecosystem Stewardship, Royal Botanic Gardens, Kew, Richmond, TW93DS, United Kingdom; Institut Botànic de Barcelona (IBB), CSIC-CMCNB, Barcelona, 08038, Spain

**Keywords:** Mycorrhiza, soil, nutrition, forest, carbon, nitrogen, phosphorus, tipping point, change, policy

## Abstract

At a time when we count on northern hemisphere forests to mitigate global atmospheric change, European forests are showing deteriorating aboveground nutritional trends without a mechanistic, causal explanation. The increasingly recognized roles of ectomycorrhizal (EM) fungi in global carbon (C), nitrogen (N), and phosphorus (P) cycling mean there is a need to understand dynamics in changing EM forests, particularly at large scales over time. Achieving this requires integrating soil microbial biology with long-term forest monitoring, and a fundamental distributional, temporal, and mechanistic understanding of key soil organisms and the plasticity of their traits across gradients. We postulate that changing abundances of ectomycorrhizas with different capabilities for delivering mineral nutrients from soil to trees, and for storing or releasing soil C, can explain what is happening with forest nutrition, and thus should be included in future models of forest nutrient cycling, above and belowground. Here, we discuss the state-of-the-art regarding data needs, focussing on environmental change, large-scale spatial and temporal dynamics, experimentation, modelling, and monitoring. Linking understanding of tree nutritional status with the potential of forests to cope with environmental change, for instance, anthropogenic C and N fertilization of the biosphere leading to P limitation, holds significant potential to inform management and policy of forests and soils for promoting resilient ecosystems.

## Graphical Abstract

**Graphical Abstract:**
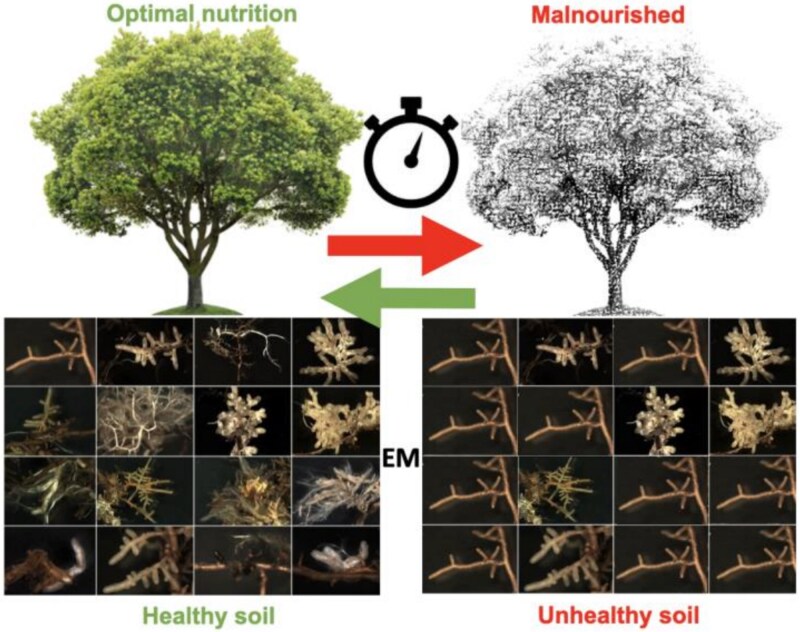

## Introduction

Since the Industrial Revolution, air pollution has been rising due to human activities. In temperate terrestrial ecosystems, nitrogen (N) primarily limits productivity [1]; across Europe, continuously high N pollution, combined with increasing CO_2_ in the air, increasing temperatures, and longer growing seasons, can result in up to 15%–20% greater forest net primary productivity [2] and, under some scenarios, increased soil carbon (C) sequestration [3]. However, this stimulatory response may be transient, and interactive effects are still poorly known. Under conditions of drought, N deposition may enhance tree mortality [4, 5]. Carbon dioxide may increase productivity aboveground at the expense of C sequestration by stimulating nutrient mining of soil organic matter [6] but may also constrain tree growth with beneficial effects on microbial necromass [7]. Higher temperatures increase photorespiration, lowering the efficiency of photosynthesis, and coincident drought can hamper the acquisition of phosphorus (P). In fact, atmospheric N deposition can eventually lead to P limitation, shifting which nutrient limits photosynthesis [8, 9]. When the soil supply of nutrients other than N is insufficient to meet the demands of faster-growing trees, their mineral nutrition deteriorates, with increased and imbalanced leaf and needle N:P ratios [10]. “Alarming” and “pervasive” nutrient imbalances outside the optimal ranges have been reported in 30% of intensively monitored forest plots across Europe [11–15] for the major forest trees, oak (*Quercus*), beech (*Fagus*), pine (*Pinus*), and spruce (*Picea*)—all forming nutritional symbioses with ectomycorrhizal (EM) fungi. Ectomycorrhizal fungi are multicellular soil microbes, obligate biotrophs that dominate soil microbial biomass, necromass, and functions in Europe’s forests, and they are responsible for up to 80% of tree productivity, N, and P acquisition [16]. These fungi can control the massive pumping of tree C into soil, and suppress its release, driving global soil C sequestration and, in some cases, mitigating CO_2_ increase [17–22]. These fungi are emerging not only as controllers of nutrient uptake by most trees but also of C dynamics in soil, mediating the impact of trees on ecosystem function [17, 21, 22]. In fact, mycorrhizas act as the nutritional nexus between plants and soil, ultimately linking atmosphere and soil. Ectomycorrhizal trees are particularly abundant in Europe [23], and up to 56% of European forests are reaching and crossing nutritional thresholds [12], without evidence so far of large-scale tree nutritional recovery or growth responses in cases of decreasing N deposition [24, 25]. The emerging nutritional limitations to tree growth can damage tree health, thereby lowering resilience to stressors such as drought, diseases, and pests, impairing forest regeneration, C storage and N retention capacities, and diminishing our ability to reduce CO_2_ emissions from fossil fuels through increased use of wood for energy [12, 26–29]. Deteriorating tree nutritional trends fundamentally compromise the sustainability and resilience of our existing and planned forests, including their environmental and economic roles [30, 31].

This deteriorating situation matters, particularly now, because forests are currently the main means for terrestrial C sequestration to mitigate climate change and most of this C is stored in the soil [32] where mycorrhizal mycelium, and in particular the mycelium of EM fungi, is a major global C pool [22]. Furthermore, densely forested areas in Europe that are efficient for large-scale C storage (e.g. Scandinavia) might harbour unique fungal communities, adapted to low temperatures and near-zero pre-industrial N deposition levels, and therefore more susceptible to drastic environmental change [33, 34]. Thus, we urgently need to understand how the fungal nexus between the atmosphere and the soil changes.

There is evidence from macroscale spatial gradients [35] indicating that EM fungi shift sharply in both taxonomic and functional composition across Europe, in concert with increasing atmospheric N pollution and deteriorating tree nutritional status. This suggests the existence of a tipping point in forests whose consequences include threats to ecosystem C and P cycling, and long-term changes to biodiversity [31]. It is much easier to recover some ecosystems before they cross tipping points than after [36], when recovery might take more effort and time, or it might not happen. Trees showing leaf or needle nutritional imbalances have different EM fungi with different functional traits in symbiosis with their roots, like species with less extraradical mycelium and with less ability to acquire nutrients in organic form [35, 37, 38]. In addition to leaf litter with altered quality entering the forest floor, reduced EM fungal growth and necromass in the soil would impact total mycelial contributions to the C cycle. Therefore, it is likely that changing abundances of EM fungal species, with different capabilities for taking up and delivering nutrients in mineral and/or organic forms from soil to trees, and for storing plant C in soil, can explain the documented imbalances in tree nutrition. Increased foliar N:P content ratios indicate European forest systems shifting from N- to P-limitation, with consequences for ecosystem health and mycorrhizal community resilience [38].

Aboveground foliar and underground fungal–root processes are directly linked, but there are research gaps in observing, quantifying, and predicting belowground processes. These gaps have implications for trees and forests, affecting our ability to understand and manage forest health and ecosystem functions like C sequestration, nutrient cycling, and ultimately, human wellbeing. Large-scale experiments with both N and P along wide environmental gradients, crossing the thresholds of N:P change in tree nutritional balance, would help address some of those gaps, but we still lack them. Such studies would vastly improve our understanding of how EM species and communities are interactively involved in the C, N, and P cycles and allow us to predict subsequent ecosystem responses and resilience to change. In this review, we discuss the data needs of decision-makers and the roles of EM fungi in shaping nutrient dynamics, forest resilience, and ecosystem tipping points in European forests, integrating perspectives from soil biology, long-term monitoring, and ecosystem modelling. We present knowledge gaps and the challenges to observe, measure, and predict belowground changes in EM communities affecting forests aboveground (Table 1). We discuss the difficulties in large-scale, multi-nutrient studies to unravel functions in EM communities and the reasons, challenges, and ways to incorporate EM fungi in models to predict forest condition and change. Moreover, we argue for the inclusion of EM fungi in long-term environmental monitoring, on a par with animals and plants. Even though we focus on Europe, given the legacy of EM studies, forest monitoring, and research on N pollution, we aim to cover topics that span global spheres of atmospheric pollution impacts on forest ecosystems and propose ways to address current knowledge gaps.

**Table 1:** Outstanding questions and knowledge gaps in understanding change in ectomycorrhizal (EM) forests, with approaches to address them.

Questions and knowledge gaps	Approaches
*How does the EM nexus between the atmosphere and the soil change over space and time?*	Long-term, spatially comprehensive belowground datasets, with structured, repeated surveys from local to continental scales.
*To what extent do EM community composition, structure, and phenotypic plasticity regulate variation in N and P supply without incurring imbalances in tree nutrition?* Understanding the roles of EM fungi in ongoing forest nutritional shifts: (i) whether and how EM temporal changes are linked with tree, soil, and forest condition changes; (ii) functional trait plasticity versus community composition shifts in relation to nutrient availability; and (iii) mechanisms of EM functional change.	Large-scale experiments with both N and P along wide environmental gradients, crossing thresholds of N:P change in tree nutritional balance. Nutrient amendment experiments (e.g. amended mycelial and/or root ingrowth bags) *In situ* meta-omics. *In situ* exoenzymatic activity assays. *In situ* hyphosphere exudate analysis. Tree nutritional and physiological status monitoring. Isotope analyses.
Observing temporal change. *Is the ability to show phenotypic variation fixed within species and/or genera, thus requiring community turnover for resilience to change?*	Standardized methods. Multiple sampling periods to detect change. Studying active communities and their traits *in situ*. Setting up a baseline against which to assess change.
Quantifying change. *Up to what point can there be system optimization instead of deterioration?*	Standardized methods that allow for data integration. *In situ* observation. Environmental and biological data collected simultaneously. Detection of active fungi. Meta-omics. Enzymatic activity analyses. Isotopic analyses. PLFAs, ergosterol Community and key-species qPCR.
Predicting change *Could the plasticity of traits make taxa resilient to change, until a point where a critical threshold is crossed, beyond which community turnover predominates?*	Sufficient replication. Data in the full range of species niche. Models including type, abundance, community composition and traits, full soil food webs, climate, soil, anthropogenic, and tree host factors. Data on which parameters change at the community level and at what speed. Models including at least one mycorrhizal type where parameters are calibrated in function of informative ecosystem data. Models including P.
*Moving from baselining to monitoring; how can we embed EM fungi in long-term, large-scale forest monitoring?*	Identify and engage policy customers. Present proof-of-concept and case studies at relevant scale(s). Build fungal science and conservation into forest monitoring to inform policy.

## Data to support decision-makers

Understanding belowground biological changes in our forests has untapped potential beyond science to inform environmental policy, forest management, and conservation efforts [24, 39]. In fact, long-term, spatially comprehensive belowground datasets, especially ones from structured, standardized, repeated surveys, are the key limiting factor in global change research, as advocated by other scientists. Such datasets, together with manipulation experiments [40], will provide a causal and mechanistic understanding of change over time in forests. Priority must be placed on building these datasets, as well as working with what currently exists. As data are collected, support is needed to follow through across multiple sampling periods, to provide the temporal dynamics necessary for “then-now-future” comparisons. Additionally, although openly available environmental or fungal data sources are valuable for large-scale ecological modelling (e.g. [41]), datasets are more scientifically valuable when both environmental and biological data are simultaneously collected in a structured, systematic way.

The UNECE ICP Forests (International Co-operative Programme on Assessment and Monitoring of Air Pollution Effects on Forests; http://icp-forests.net) network has generated, for 40 years, forest health data that are key to monitoring efforts that impact policy. Monitoring of European trees and understory vegetation led to significant findings (e.g. [42–44]) that underpinned the advancement of air pollution abatement measures in Europe and are foundational to forest knowledge, providing scientific information for the Convention on Long-Range Transboundary Air Pollution, National Emission Reduction Commitments Directive, Forest Europe, Convention on Biological Diversity, United Nations (UN) Framework Convention on Climate Change, UN Food and Agriculture Organization (UN-FAO) Global Forest Survey, Eurostat, and national policy-making bodies.

Despite their criticality to forest ecosystem health and resilience, fungi are not yet included in ICP Forests monitoring. However, fungal science and conservation can be built into ICP Forests and lead to useful insights for policy. Research has linked belowground EM assessment to ICP Forests’ long-term, intensively-monitored plots, through a belowground baseline survey of ca. 40,000 ectomycorrhizas in 137 forests across 20 European countries [35], and more recently from soil fungal environmental DNA (eDNA) from 238 forests across 15 countries [45]. The resulting knowledge enhances the relevance of science and conservation analyses, for instance, by refining critical forest nutrient pollution loads [35]. Evidence for EM plasticity in nutrient foraging traits reflects species-specific variance in fungal adaptation and potential recovery from environmental pollution [35] and has allowed understanding of links between ECM communities and tree growth, and consequently forest productivity, with a three-fold difference between fast-tree growth EM fungi communities versus slow-growth ones [46]. In addition, baseline EM data allowed generation of new evidence on EM ecoregion boundaries in Europe [33], environmental niches [47], and predicted distributional changes [48]. However, a major gap exists: belowground EM data have yet to be used for testing whether, and how, EM fungi change over time, and how any temporal changes are linked with tree, soil, and forest condition changes (Table 1). Such analyses could, uniquely, inform ectomycorrhizal forest change from local to continental scales. In fact, other forest monitoring networks covering different geographical scales and forest types, such as the ForestGEO network (https://forestgeo.si.edu), the Forest Inventory Analysis—FIA (https://research.fs.usda.gov/programs/fia), the Global Ecosystems Monitoring GEM network [49], the SEOSAW network (https://seosaw.github.io), or the ForestREplot network (https://forestreplot.ugent.be/) represent excellent research platforms for the inclusion of mycorrhizal fungi that would greatly enhance current knowledge of forest ecosystem functioning globally.

There are methods to detect mycorrhizal fungi that do not require root analysis, such as the deployment of mycelial ingrowth bags [50] or the collection of bulk soil samples for eDNA analyses. These methods offer complementary views of mycorrhizal communities, but they have limitations. For instance, not all fungi seem to enter the mycelial bags [51], and in soil, detection of DNA from dormant spores or dead fungi can lead to bias in community composition (e.g. [52]). Moreover, assessing relative abundances using semi-quantitative DNA analysis, such as high-throughput sequencing methods, is challenging. Despite being more destructive (although damage to the tree and fungi is limited to a few fine roots) than other methods, sampling fine roots and individual ectomycorrhizas has several advantages over these methods, among them the quantification of relative abundance, the detection of active communities, and direct observation of traits in ectomycorrhizas. Moreover, being longer lived than extraradical mycelium, ectomycorrhizas are potentially less seasonal than mycelium in soil and in ingrowth bags.

## Roles and status of ectomycorrhizas

Nutrient cycling in Europe’s forests is dominated by ectomycorrhizas. Fine tree roots are fully enveloped by fungal tissue, forming highly polyphyletic, diverse symbioses that mediate tree nutrient uptake and C storage in soil. Whereas many EM fungi can assimilate nutrients in soil solution in both inorganic and some organic forms, like amino acids and amino sugars [53, 54], others can access nutrients, mostly N and P, bound in complex organic compounds, like protein, chitin, and phytate [55, 56], and some even in recalcitrant organic matter like lignin and phenol complexes [57–59], and then directly transfer N and/or P to their plant hosts [60, 61]. For scavenging or mining, EM fungi can use hydrolytic exoenzymes, like phosphatases and proteases, or oxidative enzymes, like laccases and peroxidases. These enzymatic capabilities show lineage-specific genomic patterns [62], but their expression can vary among species within dominant genera like *Russula* or *Lactarius* [63] and can be influenced by environmental factors like soil pH and C:N ratio [64, 65]. However, few studies have focused on direct measurement of enzymatic capabilities of EM at different spatial scales or across environmental gradients to explore the adaptability of these fungi to nutrient availability and understand their impact on tree mineral nutrition. Standardized enzymatic assays coupled with DNA sequencing are needed to capture and properly interpret inter- and intraspecific diversity across species.

Over the last decade, it has been shown that EM forests store more C per unit of N than ecosystems dominated by other types of mycorrhizas and that ectomycorrhizas can regulate the CO_2_ fertilization effect by mobilizing soil N, including typically unavailable organic N, when more C is transferred to roots [17–19, 21, 22, 66, 67]. Whether these remain the case under increasing N deposition, as trees become P-limited, has not been addressed so far [24, 68], except regarding the role hyphosphere bacteria may play in increasing soil phosphatase activity [69]. Ectomycorrhizal richness and evenness decline sharply across large spatial gradients of increasing N deposition [70], with major shifts in dominant EM showing different functional growth, and potentially necromass, traits linked with changes in tree foliar N:P [35, 38] and tree growth [46]. Thresholds for EM fungal community change were identified at foliar N:P ratios values of 10.2 and 13.3, both values within the critical foliar N:P values for dominant tree species in Europe [12]. Thus, we can hypothesize that large-scale EM changes impact forest condition and functions through tree–fungal feedback loops [38]. To address future change, it is critical to understand spatiotemporal trends (Table 1).

## Ectomycorrhizal change over space

Most variation in EM diversity and distribution at large geographic scales had remained unexplained by environmental models due to reliance on modelled variables, non-standardized sampling, inappropriate and/or indirect assignments of EM status and traits for fungi, semi-quantitative analysis of short DNA reads to infer abundances, mixing active fungi and dormant spore banks in eDNA analysis, and/or focusing on aboveground reproductive structures that inaccurately reflect active communities belowground [35, 71–76]. Using intensive and extensive sampling of roots and environmental data, with 38 biotic and abiotic environmental variables, was paramount for the first large-scale belowground spatial EM analysis [35]. This long-term and *in situ* tree host, soil, and environmental dataset, with standardized sampling and direct analysis of individual roots, and sequencing longer DNA reads, is considered robust [77, 78] and has been scaled up successfully across Europe’s forests to generate the unbiased, large-scale, molecular, ecosystem-level baseline EM data long wished for by environmental scientists [79–82]. These EM baseline data can be used for the historical benchmarking that underlies all global change science, combined with targeted field experimental design directly guided by large-scale microbial field data, hitherto notoriously unavailable, and cutting-edge metagenomics and metatranscriptomics approaches in fungal ecology [83, 84]. Similarly, studies covering multiple plant niche compartments, such as leaves, roots, and soil at a large scale (e.g. [85]) using standardized methods, can add information about other fungal guilds and ecosystem functions. We therefore propose using the information from these large spatial studies to design studies of temporal change and environmental threshold crossings, ideally with direct assignment of mycorrhizal traits, instead of inferences from incomplete trait databases.

Most studies that measure EM functions have been carried out in microcosms or laboratory experiments, using a few easily culturable fungi; a synthesis [86] shows that measuring EM functions in the field involves challenges regarding methodology, complexity, and logistics that new approaches can tackle. Metagenomic DNA sequencing can be used to estimate changes in functional roles in EM fungi (e.g. genes involved in N and P foraging strategies) across space and time [66]. Metatranscriptomic analysis targeting gene families involved in key biogeochemical processes (e.g. acidic phosphatases for P metabolism, glutamine synthetase for increasing N limitation, oxidative enzymes such as Mn-dependent oxidases impacted by N, proteases for proteolytic activity, and phytases for phytate degradation) and markers of fungal growth (e.g. glucan synthase for fungal cell wall synthesis) can explain links between response and effect traits at the community level, model changes in effect traits to predict ecosystem processes, and assess changes in response traits to predict EM community dynamics under environmental change. For example, through their oxidative enzymes, EM fungi like *Cortinarius acutus* (currently known as *C. aurae*) can negatively impact soil C stocks [87]. Largely restricted to microcosms so far, isotope labelling experiments can also inform carbon and nutrient transfer between symbionts. Combining DNA sequencing, shotgun metagenomics, metatranscriptomics, enzymatic essays, exudation analysis, and isotope labelling of roots, soils, and mycelial samples can offer complementary views of EM diversity and function in the field and allow presence, distribution, host preference, and function to be inferred directly (Table 1). Meta-omics could help generate the understanding necessary to allow predicting how functionally key soil organisms change, persist, and recover in the face of anthropogenic environmental changes in ecosystems, both spatially and temporally, particularly when integrated with targeted field measurements and improved taxonomic and functional reference resources.

## Ectomycorrhizal change over time?

Changes in community composition through different timescales, their nature and quantitative importance are central to understanding ecosystem impacts, including C and nutrient cycling [88–90]. At small scales, we know that soil fungal communities can change slowly over time [91]. But so far, it had not been possible to study temporal change in fungi and the environment within soil at large scales, observationally or experimentally, due to the cryptic lifestyle of soil fungi and the lack of a standardized large-scale baseline. Nevertheless, historical taxonomic reliance on fungal fruitbodies for identification fuelled fungarium and citizen-science opportunistic fruitbody surveys that have provided an alternative approach to study certain fungi at broad scales (i.e. analyses of aboveground fruitbodies from historical data over the last few decades). Phenological changes have demonstrated, particularly for central and northern Europe, that sexual reproduction has changed markedly with time, and this change is often linked to air pollution and climate [92–97]. Work in the Netherlands [98] has shown that aboveground fruitbody production by some N-sensitive EM fungi (e.g. species of *Cortinarius, Tricholoma, Hydnellum,* and *Sarcodon*) declined until the 1990s, and partly recovered since then, possibly because of a decrease in N pollution.

Compositional change in long-term EM fruitbody data has been correlated to climate across multiple decades [99] and fruitbody assemblages across central to northern Europe demonstrate shared trends in similar ecoregions (e.g. boreal forests; [100]). However, whether changes in aboveground fungal reproduction reflect changes in diversity, activity, biomass, necromass, or distribution of belowground EM fungi remains less known, and ca. 40% of European EM fungi do not fruit aboveground [35]. Furthermore, neither historical aboveground reproduction datasets where decades of data are, most often, aggregated [97], nor single snapshots from belowground surveys, where changes over time are inferred from spatial gradients [35, 70, 101], provide direct evidence of large-scale EM temporal change in community structure or function, something that has long been available for animals and plants [102, 103]. Thus, there is a major knowledge gap for terrestrial ecosystem science, with the dynamics of a keystone functional guild essentially representing a “black box.”

Mycorrhizas are also considered the next frontier in achieving a whole-tree perspective on performance [104]. Aboveground tree condition changes observed across European forests, using ICP Forests foliar survey data, could be causally linked with EM changes belowground. There are reports of negative trends in foliar nutrition (contents, concentrations, and ratios) for pine, spruce, beech, oak, and other trees [11, 13, 14]; changes in litter quality [105]; and of rising defoliation and leaf discolouration in trees as N deposition increases, even under N-limiting conditions [10, 15]. An increased sensitivity to drought and ectomycorrhizal changes are proposed as explanatory mechanisms in most of the studies above—the latter mechanism being expected, given that ectomycorrhizas account for most tree productivity, N and P acquisition—but all these mechanisms remain a matter of speculation, due to a lack of temporal, large-scale EM data (Table 1).

Phosphorus limitation in EM-dominated forests is a new condition, particularly in Europe, that requires understanding. Globally, there are few naturally P-limited, EM-dominated forest ecosystems (exceptions involve woodlands dominated by dual arbuscular mycorrhizal and EM trees, largely in Africa and Australia). So far, there is little evidence that, under N enrichment, tree foliar P resorption efficiency increases, or that upregulation of phosphatases is less than the reduction in EM fungi abundance [68]. A recent meta-analysis [106] shows decoupling of N and P resorption under global warming, drought, and N deposition (with reduced N resorption and increased P resorption); moreover, elevated CO_2_ strongly reduced P resorption with no effect on N resorption. Nonetheless, naturally N-enriched microsites (e.g. animal latrines, carcasses, disturbances) may have pre-adapted some EM fungi such as *Scleroderma* and *Tomentella,* to postindustrial levels of N-eutrophication [35] and/or P-limitation. Many questions remain unanswered. To what extent might such pre-adaptation be broadly found? How could these fungi influence compositional shifts across time in response to changing forest N and P, and what are the consequences for the C cycle? *Alnus* forests, characterized by highly specialized EM communities [107] and their high demand for P to sustain N-fixation, could be ideal systems to study EM fungal P specialists.

**Figure 1:**
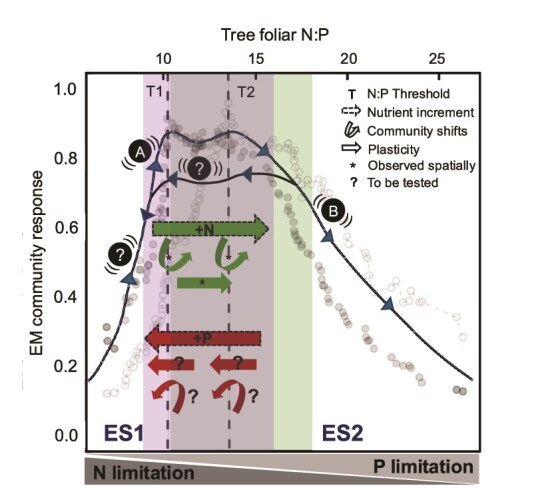
Linking changes in ectomycorrhizal (EM) communities and tree foliar nutrition. Small circles show proportions at each of 137 monitoring plots for ectomycorrhizas with significantly contrasting relationships (dark grey, mostly decreasing vs. light grey, mostly increasing) with tree foliar N:P ratios [35, 38]. There are EM composition shifts at increasing foliar N:P ratios at two thresholds (T1 and T2). The potential for change leading to a tipping point is indicated by the curve and the green area (tipping point domain). When phenotypic plasticity occurs in EM communities, lagged responses delay the forest tipping point. Changes in EM communities (A to B) lead to a tipping point in the ecosystem that shifts to an alternative state (from nutritionally balanced to nutritionally imbalanced trees, ES1 to ES2). Large circles with letters represent expected EM communities after experimental additions of N or P. With increasing foliar N:P, phenotypic EM plasticity occurs between tree nutritional thresholds, whereas EM community shifts happen when crossing thresholds (tipping points). With decreasing foliar N:P ratios, phenotypic plasticity occurs, but species replacement lags after crossing thresholds. This leads to alternative states, and hysteresis, in EM communities at similar foliar N:P. Balanced foliar N:P ratios averaged across four tree species [12] are indicated in purple.

What we do know is that sudden shifts in contrasting ecosystem states after crossing thresholds of environmental variables represent tipping points. Although there is an extensive and growing literature for aquatic and aboveground systems, with clear predicted response and effect traits [36, 108], little is known belowground [109]. Recently, a spatially inferred, putative tipping point in EM communities was linked to N deposition and tree foliar N:P ratios [38]. This is of concern because it represents the crossing of environmental and nutritional thresholds, with potentially irreversible shifts leading to alternative stable states in forest ecosystems (Fig. 1). We hypothesize that in temporal tipping points, EM fungal communities are likely to change more abruptly than in the spatially inferred tipping point domain, as replacement of species might not occur on the site due to spore bank or dispersal limitations. A tipping point in this case would lead to a steep EM community change, as well as a greater decrease in EM species diversity. Only temporal (time-series) EM data and controlled experiments can address this, as well as help understand future consequences.

## Understanding mechanisms of change

There is still limited information on how N-deposition-induced P limitation is driven by changes in EM communities, the role of species trait plasticity, and how it impacts soil C [53]. There have been numerous modelling, observational, and experimental studies at local scales of EM and emerging P limitation in Europe [110–118] and China [69] but based on forests well above the N deposition thresholds for drastic changes in EM communities [35]. Therefore, so far, studies have focussed on severely negatively N-impacted EM communities. In fact, it is unclear if there are consistently dominant, potentially nitrophilic, P-specialist EM fungi that merit physiological investigation, and how their traits would respond to shifts in both N and P limitation [68, 112, 117–123].

Ectomycorrhizal fungi show different functional traits (hyphal growth and rhizomorph formation) that may confer different abilities to mobilize inorganic and/or organic P. For instance, EM fungi with medium- and long-distance soil exploration abilities are thought to be more efficient in P mobilization than short-distance or contact explorers, due to their extended exploration of soil and exoenzymatic capabilities [124]. Nonetheless, their study in nature is largely lacking [119, 125] and we do not know if these correlations between morphological and physiological (enzymatic activity) are causally linked. Linking taxonomic with functional trait data at large scales has been a widely recognized challenge [82], and most studies linking EM communities to forest dynamics through fungal traits use traits from databases such as DEEMY [126], FUNGuild [127], and FungalTraits [128] with only a small subset of species currently represented and assuming traits are conserved at the genus level or above. However, across Europe, there are EM fungi that display intraspecific phenotypic variation in their functional growth traits (e.g. presence/abundance of hyphae and rhizomorphs), and that variation is linked to environmental conditions [35]. Large changes in effect traits at the community level may lead to tipping points in forests by, for instance, controlling the extent of nutrient uptake by EM fungi based on the distances they grow away from tree roots into the soil [38], and whether intraspecific trait variation could reinforce or counteract these community changes is unknown.

Trait variation within species and/or communities can affect their responses to environmental stress, stabilizing or destabilizing ecosystem states, with potential consequences for ecosystem recovery [31, 36, 38]. Understanding how functional traits, such as those of ectomycorrhizas affect ecosystem properties remains a key problem to resolve [129]. It is uncertain how individual EM species and communities might recover from eutrophication and nutritional imbalances in soils and trees, or how resilient and plastic EM communities are to change over time. After a decrease in N addition to the system, there are three potential scenarios (Fig. 1), as proposed for grassland plants [130]: (i) instant species richness recovery (no hysteresis); (ii) lagged responses at species/community level, influenced by phenotypic plasticity and the cumulative effect of long-term N addition (ecological hysteresis); and (iii) irreversible changes in communities (e.g. due to limited dispersal abilities and propagule depletion (e.g. spore banks)—fundamental regime shift). The functional trait plasticity of EM is likely linked to tipping points and tree nutritional status (Fig. 1); scenarios will depend on species composition, genetic diversity, site history, and conditions along the environmental gradient.

A key challenge is how to inject reality into environmental models limited by poor soil mechanistic information [115], and micro- or mesocosm physiological ecology experiments limited by ongoing reliance on a few axenically culturable EM fungi (that are hardly representative of forest EM communities; [82]). Because some forest areas are showing signs of partial recovery due to declining nitrogen deposition resulting from national regulatory policies [131], while others are shifting from N to P limitation [12], it would be possible to compare changes in ectomycorrhizas at forest sites with deteriorating, stable, or improving tree conditions, as inferred from foliar N:P ratios. Field experimentation can close the knowledge gap that has arisen from over-reliance on easy-to-manipulate fungi instead of those that dominate forest soil [82]. Furthermore, due to lack of baseline information and a strong plant-centric bias, EM field experiments have traditionally used host tree species to select field sites, instead of considering what fungi are present. A pervasive disconnect between lab and field has resulted, alongside frequent proposals to bring hard-to-manipulate field fungi into experimental micro- or mesocosms that have had limited success. Focussing directly on a range of known field EM communities for experiments across Europe’s sharp environmental gradients can allow to understand how EM contribute to N, P, and C dynamics (Table 1).

Ectomycorrhizal fungi interact with many other soil organisms, including other fungi, microeukaryotes, and prokaryotes, as well as micro- and macrofauna, and these interactions can affect forest dynamics. Ectomycorrhizal fungi interact with arbuscular (AM) mycorrhizal fungi, which are associated with common trees like rowan (*Sorbus aucuparia*), yew (*Taxus baccata*), or maple (*Acer* spp.), and most forest understory plants. In fact, due to the different nutrient acquisition strategies and necromass of these fungi, and their tree host litter properties, AM- and EM-dominated forests exhibit different C, N, and P dynamics [132, 133]. Some forest understories are dominated by ericoid mycorrhizal plants, such as heathers, whose fungi show extensive C-degrading enzymatic repertoires [134]. Ectomycorrhizal fungi may compete with fungal saprotrophs (i.e. wood and litter decomposers), slowing down decomposition and therefore decelerating C cycling, but this “Gadgil effect” is dependent on litter quality and EM fungal community composition [135]. They also interact with bacteria, which can positively affect plant and fungal growth [136], as is the case with a group of bacteria (mycorrhizal helper bacteria) that can enhance the mycorrhizal symbiosis and suppress plant defence [137, 138]. Both AM and EM fungi can harbour bacteria on their hyphae, which can release phosphatases and organic acid anions to mobilize soluble inorganic and organic P, enhancing plant P uptake [59, 139]. We can expect soil biotic interactions to modulate change in EM forests, justifying investigation of the EM hyphosphere biota, including via field experimentation and forest modelling.

## Forest mycorrhizas and biosphere models

Models describing mycorrhizal fungal growth and the interaction between mycorrhizas and a single plant are relatively common (reviewed by [140, 141]). However, most terrestrial biosphere models and ecosystem models do not include explicit representation of mycorrhizas. Some ecosystem/biosphere models are working on integrating representations of mycorrhizas, as well as a few other soil functional groups (e.g. bacteria, fungivores), such as the Community Land Model (CLM), the current trunk version of Organizing Carbon and Hydrology in Dynamic Ecosystems (ORCHIDEE), Yasso [142], and QUINCY (based on CENTURY, [143]). Using Yasso, including mycorrhizal type, yields better outcomes concerning litter decomposition in EM-dominated versus AM-dominated ecosystems [142]. QUINCY [143] shows how including mycorrhizas better represents C and N dynamics because, without them, plant N-uptake is limited to mineral N. Still, such global models have important limitations because: (i) they use an implicit approach in which those functional groups are merely included for adjusting the rates of some processes (e.g. litter decomposition); (ii) soil biotic interactions and food webs are not fully developed, with many key functions still missing; specifically, interactions between AM and EM fungi and rhizosphere bacteria, including priming mechanisms, are crucial to understanding and therefore modelling soil organic C changes [144–146]; (iii) the representation of biogeochemical cycling in soils is still dominated by chemical composition, underestimating the impacts of soil physical protection and accessibility of organic matter [147], and the ability of some organisms to use alternative sources of nutrients (e.g. decomposer abilities of some EM, [87]; and (iv) although there is generally a distinction between AM and EM in these models, no further differentiation in types, diversity, or relative abundance is possible.

The model KEYLINK [11, 141, 146, 148, 149] has been developed to address those issues by explicitly representing all the main functional groups among the soil food web, as well as their interactions with soil physical structure. Moreover, ORCHIDEE is currently being further developed to include those aspects by merging KEYLINK into it as a new branch (for details see [150]). This will offer an explicit representation of the soil food web, including mycorrhizas, combined with the rest of functional groups among global models. Different EM soil exploration types could be simulated by the coupled model, including the development of rhizomorphs and the extension of the hyphal network. Unlike most soil models where chemical recalcitrance is central to determining decay of organic matter, soil biota drive decay and cycling in KEYLINK. Mycorrhizas are modelled including their C input to soil (through turnover of C from symbiosis), decay of soil organic matter (in competition with saprotrophic fungi and bacteria), and soil aggregation [149], which, in turn, determine availability of N, both directly through mycorrhizas and through decay of organic compounds releasing nutrients in soil. Currently, the P cycle is not represented in ecosystem-scale models including mycorrhizas, which limits applicability in P-limited forests. In general, some of the main obstacles for improved model development are observational constraints, resulting in high parameter uncertainty [143]. Thus, simulating different EM and AM fungi is possible using existing modelling frameworks and average values for key parameters (e.g. turnover, rhizomorph formation, and length) of the mycorrhizal community could work at the ecosystem scale. However, changes in community composition would then need to be implemented as changes in the parameters of the mycorrhizal community, which would require clear data on which parameters change at the community level and at what speed. Even without explicit simulation of the change, models could be used to compare the ecosystem response to different communities represented by their averages. Nevertheless, to our knowledge, this has not yet been attempted.

## Monitoring forest mycorrhizas

Biological monitoring has highly developed national and international regulatory frameworks in aquatic systems, where sampling is more straightforward than in soil, leading to well-established benchmarks, metrics, and a wide range of associated research by communities of experts. In contrast, national and international soil monitoring for fungi is typically nonexistent or in its infancy [151], with only rare exceptions such as in the Netherlands and Denmark. Nonetheless, EM fungi can be large (up to 10 m) and long-lived (years to centuries), and their reproductive structures are commercially harvested and traded internationally (e.g. chanterelles, porcini, truffles). Since the 1980s, EM fungal declines have been documented within European countries [152], and declines of many forest EM fungi in northern and central European countries have been tied to N deposition [124]. One would expect organisms with the above characteristics to be common conservation targets and their monitoring to occur at the regional rather than country level, reflecting belowground species distributional changes [153, 154]. There is an urgency to develop standardized mycorrhizal protocols and metrics that can be applied at regional scales to inform public policy and forest management (e.g. as in England’s Natural Capital and Ecosystem Assessment programme, https://environment.data.gov.uk/natural-capital-ecosystem-assessment/about). Remote sensing data have been recently used to scale biodiversity science, with broad applications including the use of tree canopy spectral properties to assess plant mycorrhizal type and plant traits [155, 156] coupled with machine learning techniques and climate data to derive global maps of leaf traits [157]. Ground truthing studies linking remote sensing assessments with *in situ* mycorrhizal communities will help upscale forest ecosystem research.

## Data Availability

Data sharing not applicable to this article as no datasets were generated or analysed during the current study.

## References

[1] Elser JJ, Bracken MES, Cleland EE, et al. Global analysis of nitrogen and phosphorus limitation of primary producers in freshwater, marine and terrestrial ecosystems. *Ecol Lett* 2007;10:1135–42. 10.1111/j.1461-0248.2007.01113.x 17922835

[2] Rehfuess KE, Ågren GI, Andersson F, et al. Relationships between Recent Changes of Growth and Nutrition of Norway Spruce, Scots Pine, and European Beech Forests in Europe: Recognition. Finland: European Forest Institute, 1999.

[3] Janssens IA, Dieleman W, Luyssaert S, et al. Reduction of forest soil respiration in response to nitrogen deposition. *Nat Geosci* 2010;3:315–22. 10.1038/ngeo844

[4] Magill AH, Aber JD, Currie WS, et al. Ecosystem response to chronic nitrogen additions. *For Ecol Manag* 2004;196:7–28. 10.1016/j.foreco.2004.03.033

[5] Allen MF, Allen EB, Lansing JL, et al. Responses to chronic N fertilization of ectomycorrhizal piñon but not arbuscular mycorrhizal juniper. *J Arid Environ* 2010;74:1170–6. 10.1016/j.jaridenv.2010.05.001

[6] Terrer C, Phillips RP, Hungate BA, et al. Trade-off between plant and soil carbon storage. *Nature* 2021;591:599–603. 10.1038/s41586-021-03306-8 33762765

[7] Franklin O, Näsholm T, Högberg P, et al. Forests trapped in nitrogen limitation. *New Phytol* 2014;203:657–66. 10.1111/nph.12840 24824576 PMC4199275

[8] Gress SE, Nichols TD, Northcraft CC, et al. Nutrient limitation in soils exhibiting differing nitrogen availabilities. *Ecology* 2007;88:119–30. 10.1890/0012-9658(2007)88[119:NLISED]2.0.CO;2 17489460

[9] Goswami S, Fisk MC, Vadeboncoeur MA, et al. Phosphorus limitation of aboveground production in northern hardwood forests. *Ecology* 2018;99:438–49. 10.1002/ecy.2100 29205288

[10] Veresoglou SD, Peñuelas J, Fischer R, et al. Exploring continental-scale stand health – N:P ratio relationships for European forests. *New Phytol* 2014;202:422–30. 10.1111/nph.12665 24387190

[11] Jonard M, Fürst A, Verstraeten A, et al. Tree mineral nutrition is deteriorating in Europe. *Glob Chang Biol* 2015;21:418–30. 10.1111/gcb.12657 24920268

[12] Krüger I, Sanders TGM, Potočić N. Increased Evidence of Nutrient Imbalances in Forest Trees across Europe. ICP Forests Brief No. 4. Eberswalde: Programme Co-ordinating Centre of ICP Forests, Thünen Institute of Forest Ecosystems, 2020. 10.3220/ICP1597824383000

[13] Peñuelas J, Fernández-Martínez M, Vallicrosa H, et al. Declining nutritional status of European forests. *Commun Biol* 2020;3:125. 10.1038/s42003-020-0839-y 32170162 PMC7070084

[14] Du E, van Doorn M, de Vries W. Spatially divergent trends of nitrogen versus phosphorus limitation across European forests. *Sci Total Environ* 2021;771:145391. 10.1016/j.scitotenv.2021.145391 33529819

[15] Potočić N, Timmermann V, Ognjenović M, et al. Tree Health Is Deteriorating in the European Forests. ICP Forests Brief No. 5. Eberswalde: Programme Co-ordinating Centre of ICP Forests, Thünen Institute of Forest Ecosystems, 2021. 10.3220/ICP1638780772000

[16] van der Heijden MGA, Martin FM, Selosse MA, et al. Mycorrhizal ecology and evolution. *New Phytol* 2015;205:1406–23. 10.1111/nph.13288 25639293

[17] Clemmensen KE, Bahr A, Ovaskainen O, et al. Roots and associated fungi drive long-term carbon sequestration. *Science* 2013;339:1615–8. 10.1126/science.1231923 23539604

[18] Averill C, Turner BL, Finzi AC. Mycorrhiza-mediated competition between plants and decomposers drives soil carbon storage. *Nature* 2014;505:543–5. 10.1038/nature12901 24402225

[19] Terrer C, Vicca S, Hungate BA, et al. Mycorrhizal association controls CO₂ fertilization. *Science* 2016;353:72–4. 10.1126/science.aaf4610 27365447

[20] Terrer C, Vicca S, Stocker BD, et al. Ecosystem responses to elevated CO2 governed by plant–soil interactions and the cost of nitrogen acquisition. *New Phytol* 2018;217:507–22. 10.1111/nph.14872 29105765

[21] Soudzilovskaia NA, van Bodegom PM, Terrer C, et al. Global mycorrhizal plant distribution. *Nat Commun* 2019;10:5077. 10.1038/s41467-019-13019-2 31700000 PMC6838125

[22] Hawkins HJ, Cargill RI, van Nuland ME, et al. Mycorrhizal mycelium as a global carbon pool. *Curr Biol* 2023;33:R560–73. 10.1016/j.cub.2023.02.027 37279689

[23] Steidinger BS, Crowther TW, Liang J, et al. Climatic controls of decomposition drive symbioses. *Nature* 2019;569:404–8. 10.1038/s41586-019-1128-0 31092941

[24] de Vries W, Du E. Chapter 1 - nitrogen deposition and its impacts on forest ecosystems: A global perspective. In: Atmospheric Nitrogen Deposition to Global Forests. London: Academic Press, 2024, 1–13. 10.1016/B978-0-323-91140-5.00013-0

[25] Schmitz A, Sanders TG, Bolte A, et al. Chapter 13 - responses of forest ecosystems in Europe to decreasing nitrogen deposition. In: Du E., de Vries W. (eds.), Atmospheric Nitrogen Deposition to Global Forests. London: Academic Press, 2024, 227–45. 10.1016/B978-0-323-91140-5.00017-8

[26] Kint V, Aertsen W, Campioli M, et al. Radial growth change of temperate tree species in response to altered regional climate and air quality in the period 1901-2008. *Climate Change* 2012;115:343–63. 10.1007/s10584-012-0465-x

[27] Etzold S, Ferretti M, Reinds GJ, et al. Nitrogen deposition is the most important environmental driver of growth of pure, even-aged and managed European forests. *For Ecol Manag* 2020;458:117762. 10.1016/j.foreco.2019.117762

[28] Dietrich V, Lauritz M, Roggenhofer MM, et al. Drought effects on growth and density of temperate tree regeneration under different levels of nitrogen deposition. *For Ecol Manag* 2024;559:121825. 10.1016/j.foreco.2024.121825

[29] Xie D, Duan L, Du E, et al. Chapter 14 - indicators and thresholds for nitrogen saturation. In: Du E., de Vries W. (eds.), Atmospheric Nitrogen Deposition to Global Forests. London: Academic Press, 2024, 249–61. 10.1016/B978-0-323-91140-5.00021-X

[30] Bidartondo MI, Suz LM. Fungi, nitrogen deposition and forests: challenges and changes. *In Pract* 2020;110:26–30.

[31] Suz LM, Bode J, Byrne A, et al. Nutrients, carbon, mycorrhizas and tipping points in forests. *Q J For* 2022;116:36–43.

[32] Lal R . Carbon sequestration. *Philos Trans R Soc B* 2008;363:815–30. 10.1098/rstb.2007.2185 PMC2610111 17761468

[33] Delhaye G, van der Linde S, Bauman D, et al. Ectomycorrhizal fungi are influenced by ecoregion boundaries across Europe. *Glob Ecol Biogeogr* 2025;33:e13837. 10.1111/geb.13837

[34] Andrew C . Not always optimal: fungal fruiting triggers indicate climate sensitivity in cooler regions. *Fungal Ecol* 2025;75:101416. 10.1016/j.funeco.2025.101416

[35] van der Linde S, Suz LM, Orme CDL, et al. Environment and host control ectomycorrhizal fungi. *Nature* 2018;558:243–8. 10.1038/s41586-018-0189-9 29875410

[36] Dakos V, Matthews B, Hendry AP, et al. Ecosystem tipping points in an evolving world. *Nat Ecol Evol* 2019;3:355–62. 10.1038/s41559-019-0797-2 30778190

[37] van der Linde S, Suz LM, Cox F, et al.Nitrogen deposition changes ectomycorrhizal fungi. In: Seidling W. (ed.), Forest Conditions. Mertinkat, Eberswalde: ICP Forests Executive Report, 2018, 9–10.

[38] Suz LM, Bidartondo MI, van der Linde S, et al. Ectomycorrhizas and tipping points. *New Phytol* 2021;231:1700–7. 10.1111/nph.17547 34110018

[39] Suz LM, Barsoum N, Benham S, et al. Monitoring ectomycorrhizal fungi at large scales. *Ann For Sci* 2015;72:877–85. 10.1007/s13595-014-0447-4

[40] de Boeck HJ, Vicca S, Roy J, et al. Global change experiments: challenges and opportunities. *Bioscience* 2015;65:922–31. 10.1093/biosci/biv099

[41] Andrew C, Büntgen U, Egli S, et al. Open-source data reveal how collections-based fungal diversity is sensitive to global change. *Appl Plant Sci* 2019;7:e01227. 10.1002/aps3.1227 30937219 PMC6426159

[42] Seidling W, Fischer R, Granke O. Relationships between forest floor vegetation on ICP forests monitoring plots in Europe and basic variables in soil and nitrogen deposition. *Int J Environ Stud* 2018;65:311–22. 10.1080/00207230701862538

[43] van Dobben H, de Vries W. Relation between forest vegetation, atmospheric deposition and site conditions at regional and European scales. *Environ Pollut* 2010;158:921–33. 10.1016/j.envpol.2009.09.015 19811862

[44] Dirnböck T, Grandin U, Bernhardt-Römermann M, et al. Forest floor vegetation response to nitrogen deposition in Europe. *Glob Chang Biol* 2014;20:429–40. 10.1111/gcb.12440 24132996

[45] Anthony MA, Tedersoo L, De Vos B, et al. Fungal community composition predicts forest carbon storage at a continental scale. *Nat Commun* 2024;15:2385. 10.1038/s41467-024-46792-w 38493170 PMC10944544

[46] Anthony MA, Crowther TW, van der Linde S, et al. Forest tree growth is linked to mycorrhizal fungal composition and function across Europe. *ISME J* 2022;16:1327–36. 10.1038/s41396-021-01159-7 35001085 PMC9038731

[47] Qi M, Suz LM, Bidartondo MI, et al. Fruitbody and root data infer niches for ectomycorrhizal fungi. *J Biogeogr* 2024;51:2221–36. 10.1111/jbi.14986

[48] Qi M, Bidartondo MI, Suz LM, et al. Predicted effects of climate change on ectomycorrhizal fungi. *Ecol Evol* 2025;15:e72743. 10.1002/ece3.72743 41426638 PMC12711597

[49] Malhi Y, Girardin C, Metcalfe DB, et al. Global ecosystems monitoring network. *Biol Conserv* 2021;253:108889. 10.1016/j.biocon.2020.108889

[50] Wallander H, Nilsson LO, Hagerberg D, et al. Estimation of the biomass and seasonal growth of external mycelium of ectomycorrhizal fungi in the field. *New Phytol* 2001;151:753–60. 10.1046/j.0028-646x.2001.00199.x 33853251

[51] Kjøller R . Disproportionate abundance between ectomycorrhizal root tips and their associated mycelia. *FEMS Microbiol Ecol* 2006;58:214–24. 10.1111/j.1574-6941.2006.00166.x 17064263

[52] Carini P, Marsden PJ, Leff JW, et al. Relic DNA is abundant in soil and obscures estimates of soil microbial diversity. *Nat Microbiol* 2016;2:16242. 10.1038/nmicrobiol.2016.242 27991881

[53] Lilleskov EA, Kuyper TW, Bidartondo MI, et al. Chapter 16 - impacts of nitrogen deposition on forest mycorrhizal communities. In: Du E., de Vries W. (eds.), Atmospheric Nitrogen Deposition to Global Forests. London: Academic Press, 2024, 95–118. 10.1016/B978-0-323-91140-5.00001-4

[54] Smith SE, Read D. In: Smith S.E., Read D. (eds.), Mycorrhizal Symbiosis (Third Edition). London: Academic Press, 2008.

[55] Bending GD, Read DJ. Activities of nutrient mobilizing enzymes in birch litter colonized by *Paxillus involutus*. *New Phytol* 1995;130:411–7. 10.1111/j.1469-8137.1995.tb01835.x

[56] Read DJ, Perez-Moreno J. Mycorrhizas and nutrient cycling. *New Phytol* 2003;157:475–92. 10.1046/j.1469-8137.2003.00704.x 33873410

[57] Lindahl BD, Tunlid A. Ectomycorrhizal fungi – potential organic matter decomposers. *New Phytol* 2014;205:1443–7. 10.1111/nph.13201 25524234

[58] Shah F, Nicolás C, Bentzer J, et al. Ectomycorrhizal fungi decompose organic matter. *New Phytol* 2016;209:1705–19. 10.1111/nph.13722 26527297 PMC5061094

[59] Chen Q, Strashnov I, van Dongen B, et al. Environmental dependency of ectomycorrhizal fungi as soil organic matter oxidizers. *New Phytol* 2024;244:2536–47. 10.1111/nph.20205 39417445 PMC11579442

[60] Abuzinadah RA, Finlay RD, Read DJ. Effect of proteins in the nitrogen nutrition of ectomycorrhizal plants. *New Phytol* 1986;103:495–506. 10.1111/j.1469-8137.1986.tb02887.x

[61] Näsholm T, Kielland K, Ganeteg U. Uptake of organic nitrogen by plants. *New Phytol* 2009;182:31–48. 10.1111/j.1469-8137.2008.02751.x 19210725

[62] Miyauchi S, Kiss E, Kuo A, et al. Genome sequencing of mycorrhizal fungi. *Nat Commun* 2020;11:5125. 10.1038/s41467-020-18795-w 33046698 PMC7550596

[63] Looney BP, Miyauchi S, Morin E, et al. Evolutionary transition to the ectomycorrhizal habit. *New Phytol* 2022;233:2294–309. 10.1111/nph.17892 34861049

[64] Courty PE, Buée M, Diedhiou AG, et al. Role of ectomycorrhizal communities in forest ecosystem processes. *Soil Biol Biochem* 2010;42:679–98. 10.1016/j.soilbio.2009.12.006

[65] Fernandez CW, See CR. The pH influence on ectomycorrhizal nitrogen acquisition and decomposition. *New Phytol* 2025;246:867–75. 10.1111/nph.70021 40065484 PMC11982800

[66] Pellitier PT, Ibáñez I, Zak DR, et al. Ectomycorrhizal access to organic nitrogen mediates CO₂ fertilization. *Nat Commun* 2021;12:5403. 10.1038/s41467-021-25652-x 34518539 PMC8438073

[67] Yan G, Fan C, Zheng J, et al. Forest carbon stocks increase with ectomycorrhizal dominance. *Nat Commun* 2024;15:5959. 10.1038/s41467-024-50423-9 39009629 PMC11251171

[68] Kuyper TW, Suz LM. Do ectomycorrhizal trees select fungi that enhance phosphorus uptake? *Forests* 2023;14:467. 10.3390/f14030467

[69] Zhang Z, Guo W, Wang J, et al. Extraradical hyphae alleviate phosphorus deficiency. *New Phytol* 2023;239:1651–64. 10.1111/nph.19078 37322611

[70] Suz LM, Barsoum N, Benham S, et al. Environmental drivers of ectomycorrhizal communities in Europe's temperate oak forests. *Mol Ecol* 2014;23:5628–44. 10.1111/mec.12947 25277863

[71] Gardes M, Bruns TD. Community structure of ectomycorrhizal fungi in a Pinus muricata forest. *Can J Bot* 1996;74:1572–83. 10.1139/b96-190

[72] Anderson IC, Cairney JW. Ectomycorrhizal fungi: exploring the mycelial frontier. *FEMS Microbiol Rev* 2007;31:388–406. 10.1111/j.1574-6976.2007.00073.x 17466031

[73] Talbot JM, Bruns TD, Taylor JW, et al. Endemism across the soil mycobiome. *PNAS* 2014;111:6341–6. 10.1073/pnas.1402584111 24733885 PMC4035912

[74] Tedersoo L, Bahram M, Põlme S, et al. Global diversity of soil fungi. *Science* 2014;346:1256688. 10.1126/science.1256688 25430773

[75] Glassman SI, Peay KG, Talbot JM, et al. A continental view of pine-associated ectomycorrhizal fungal spore banks. *New Phytol* 2015;205:1619–31. 10.1111/nph.13240 25557275

[76] Brundrett MC, Tedersoo L. Evolutionary history of mycorrhizal symbioses and global host plant diversity. *New Phytol* 2018;220:1108–15. 10.1111/nph.14976 29355963

[77] Buée M, Reich M, Murat C, et al. 454 pyrosequencing analyses reveal high fungal diversity. *New Phytol* 2009;184:449–56. 10.1111/j.1469-8137.2009.03003.x 19703112

[78] Tedersoo L, Lindahl B. Fungal identification biases in microbiome projects. *Environ Microbiol Rep* 2016;8:774–9. 10.1111/1758-2229.12438 27348848

[79] Lilleskov EA, Parrent JL. Can we develops predictive models of mycorrhizal fungal community–environment relationships? *New Phytol* 2007;174:250–6. 10.1111/j.1469-8137.2007.02023.x 17388888

[80] Cox F, Barsoum N, Bidartondo MI, et al. A leap forward in geographic scale for forest ectomycorrhizal fungi. *Ann For Sci* 2010;67:200. 10.1051/forest/2009107

[81] Peay KG, Bidartondo MI, Arnold AE. Scaling fungal–plant interactions. *New Phytol* 2010;185:878–82. 10.1111/j.1469-8137.2009.03158.x 20356342

[82] Zak DR, Pellitier PT, Argiroff W, et al. Role of ectomycorrhizal fungi in soil carbon dynamics. *New Phytol* 2019;223:33–9. 10.1111/nph.15679 30636276

[83] Law SR, Serrano AR, Daguerre Y, et al. Metatranscriptomics captures shifts in mycorrhizal coordination. *Proc Natl Acad Sci USA* 2022;119:e2118852119. 10.1073/pnas.2118852119 35727987 PMC9245616

[84] Auer L, Buée M, Fauchery L, et al. Metatranscriptomics sheds light on the links between the functional traits of fungal guilds and ecological processes in forest soil ecosystems. *New Phytol* 2024;242:1676–90. 10.1111/nph.19471 38148573

[85] van Nuland ME, Daws SC, Bailey JK, et al. Fungal biodiversity of *Populus* trees. *Nat Microbiol* 2023;8:2406–19. 10.1038/s41564-023-01514-8 37973868

[86] Lekberg Y, Helgason T. *In situ* mycorrhizal function – knowledge gaps. *New Phytol* 2018;220:957–62. 10.1111/nph.15064 29436724

[87] Lindahl BD, Kyaschenko J, Varenius K, et al. A group of ectomycorrhizal fungi restricts organic matter accumulation. *Ecol Lett* 2021;24:1341–51. 10.1111/ele.13746 33934481

[88] Pickett STA, Collins SL, Armesto JJ. Causes and mechanisms of succession. *Vegetatio* 1987;69:109–14. 10.1007/BF00038691

[89] McEwan RW, Dyer JM, Pederson N. Multiple interacting ecosystem drivers in oak forests. *Ecography* 2011;34:244–56. 10.1111/j.1600-0587.2010.06390.x

[90] Vellend M . The Theory of Ecological Communities (MPB-57). Princeton: Princeton University Press, 2016. 10.1515/9781400883790

[91] Martinović T, Odriozola I, Mašínová T, et al. Temporal turnover of soil microbiome composition. *Ecol Lett* 2021;24:2726–38. 10.1111/ele.13896 34595822

[92] Arnolds E . Decline of ectomycorrhizal fungi in Europe. *Agric Ecosyst Environ* 1991;35:209–44. 10.1016/0167-8809(91)90052-y

[93] Gange AC, Gange EG, Sparks TH, et al. Rapid and recent changes in fungal fruiting patterns. *Science* 2007;316:71. 10.1126/science.1137489 17412949

[94] Kauserud H, Heegaard E, Büntgen U, et al. Warming-induced shift in European mushroom fruiting phenology. *Proc Natl Acad Sci* 2012;109:14488–93. 10.1073/pnas.1200789109 22908273 PMC3437857

[95] Boddy L, Büntgen U, Egli S, et al. Climate variation effects on fungal fruiting. *Fungal Ecol* 2014;10:20–33. 10.1016/j.funeco.2013.10.006

[96] Bidartondo MI, Ellis C, Kauserud H, et al. Climate change: Fungal responses and effects. In: State of the World's Fungi. Kew, London: Royal Botanic Gardens, 2018.

[97] Andrew C, Heegaard E, Høiland K, et al. Explaining European fungal fruiting phenology with climate variability. *Ecology* 2018;99:1306–15. 10.1002/ecy.2237 29655179

[98] van Strien AJ, Boomsluiter M, Noordeloos ME, et al. Woodland ectomycorrhizal fungi benefit from reduced nitrogen deposition. *J Appl Ecol* 2018;55:290–8. 10.1111/1365-2664.12944

[99] Andrew C, Heegaard E, Halvorsen R, et al. Climate change impacts on fungal community and trait dynamics. *Fungal Ecol* 2016;22:17–25. 10.1016/j.funeco.2016.03.005

[100] Andrew C, Halvorsen R, Heegaard E, et al. Continental-scale macrofungal assemblage patterns correlate with climate, soil carbon and nitrogen deposition. *J Biogeogr* 2018;45:1942–53. 10.1111/jbi.13374

[101] Cox F, Barsoum N, Lilleskov E, et al. Nitrogen availability is a primary determinant of conifer mycorrhizas. *Ecol Lett* 2010;13:1103–13. 10.1111/j.1461-0248.2010.01494.x 20545731

[102] Lenoir J, Svenning JC. Climate-related range shifts – a global multidimensional synthesis and new research directions. *Ecography* 2015;38:15–28. 10.1111/ecog.00967

[103] Pecl GT, Araújo MB, Bell JD, et al. Biodiversity redistribution under climate change. *Science* 2017;355:eaai9214. 10.1126/science.aai9214 28360268

[104] Weemstra M, Kuyper TW, Sterck FJ, et al. Incorporating belowground traits. *Oikos* 2023;2023:e08827. 10.1111/oik.08827

[105] van Diepen LT, Frey SD, Sthultz CM, et al. Changes in litter quality caused by simulated nitrogen deposition reinforce the N-induced suppression of litter decay. *Ecosphere* 2015;6:1–16. 10.1890/ES15-00262.1

[106] Hong Y, Chen J, Lü X-T, et al. Decoupling of plant nitrogen and phosphorus under global change over the last two decades. *J Ecol* 2025;113:2120–30. 10.1111/1365-2745.70081

[107] Tedersoo L, Suvi T, Jairus T, et al. Revisiting ectomycorrhizal fungi of the genus *Alnus*: differential host specificity, diversity and determinants of the fungal community. *New Phytol* 2009;182:727–35. 10.1111/j.1469-8137.2009.02792.x 19320837

[108] Lever JJ, van de Leemput IA, Weinans E, et al. Foreseeing the future of mutualistic communities beyond collapse. *Ecol Lett* 2020;23:2–15. 10.1111/ele.13401 31707763 PMC6916369

[109] Clemmensen KE, Durling MB, Michelsen A, et al. A tipping point in carbon storage when forest expands into tundra is related to mycorrhizal recycling of nitrogen. *Ecol Lett* 2021;24:1193–204. 10.1111/ele.13735 33754469

[110] Braun S, Thomas V, Quiring R, et al. Does nitrogen deposition increase forest production? The role of phosphorus. *Environ Pollut* 2009;158:2043–52. 10.1016/j.envpol.2009.11.030 20015583

[111] Bahr A, Ellström M, Bergh J, et al. Nitrogen leaching and ectomycorrhizal nitrogen retention capacity in a Norway spruce forest. *Plant Soil* 2015;390:323–35. 10.1007/s11104-015-2408-6

[112] Rosenstock NP, Berner C, Smits MM, et al. Role of nutrient availability in fungal exploration. *New Phytol* 2016;211:542–53. 10.1111/nph.13928 26996085

[113] Zavišić A, Nassal P, Yang N, et al. Phosphorus availability shapes ectomycorrhizal communities. *Soil Biol Biochem* 2016;98:127–37. 10.1016/j.soilbio.2016.04.006

[114] Zavišić A, Yang N, Marhan S, et al. Soil phosphorus resources affect ectomycorrhizal communities. *Front Plant Sci* 2018;9:463. 10.3389/fpls.2018.00463 29706979 PMC5908982

[115] Bortier MF, Andivia E, Genon JG, et al. Towards understanding the role of ectomycorrhizal fungi in forest phosphorus cycling: a modelling approach. *Cent Eur For J* 2018;64:79–95. 10.1515/forj-2017-0037

[116] Köhler J, Yang N, Pena R, et al. Ectomycorrhizal fungal diversity increases phosphorus uptake efficiency. *New Phytol* 2018;220:1200–10. 10.1111/nph.15208 29770963

[117] Almeida JP, Rosenstock NP, Forsmark B, et al. Ectomycorrhizal community composition and function in a spruce forest transitioning between nitrogen and phosphorus limitation. *Fungal Ecol* 2019;40:20–31. 10.1016/j.funeco.2018.05.008

[118] Almeida JP, Menichetti L, Ekblad A, et al. Phosphorus regulates ectomycorrhizal fungi biomass production in a Norway spruce forest. *Biogeosciences* 2023;20:1443–58. 10.5194/bg-20-1443-2023

[119] Lilleskov EA, Kuyper TW, Bidartondo MI, et al. Atmospheric nitrogen deposition and forest mycorrhizal diversity: a review. *Environ Pollut* 2019;246:148–62. 10.1016/j.envpol.2018.11.074 30543941

[120] Maaroufi NI, Nordin A, Palmqvist K, et al. Nitrogen enrichment enhances soil carbon accumulation. *Glob Chang Biol* 2019;25:2900–14. 10.1111/gcb.14722 31166650

[121] Ruess RW, Swanson MM, Kielland K, et al. Phosphorus mobilizing enzymes in boreal fungi. *Forests* 2019;10:554. 10.3390/f10070554

[122] Zuo R, Zou F, Tian S, et al. Effects of *scleroderma* sp. and phosphate on seedling quality. *Agronomy* 2022;12:901. 10.3390/agronomy12040901

[123] Chen W, He L, Tian S, et al. The role of ectomycorrhization with *scleroderma* sp. in promoting substrate nutrients mobilization under phosphorus-enriched compost amendment: a case study with *Castanea henryi* seedlings. *For Ecol Manag* 2023;532:120823. 10.1016/j.foreco.2023.120823

[124] Lilleskov EA, Hobbie EA, Horton TR. Conservation of ectomycorrhizal fungi under nitrogen deposition. *Fungal Ecol* 2011;4:174–83. 10.1016/j.funeco.2010.09.008

[125] Plassard C, Louche J, Ali MA, et al. Diversity in phosphorus mobilisation in ectomycorrhizal fungi. *Ann For Sci* 2011;68:33–43. 10.1007/s13595-010-0005-7

[126] Agerer R, Rambold G. DEEMY – the concept of a characterization and determination system for ectomycorrhizae. *Mycorrhiza* 1997;7:113–6. 10.1007/s005720050171

[127] Nguyen NH, Song Z, Bates ST, et al. FUNGuild: an annotation tool for fungal datasets. *Fungal Ecol* 2016;20:241–8. 10.1016/j.funeco.2015.06.006

[128] Põlme S, Abarenkov K, Nilsson RH, et al. FungalTraits database. *Fungal Divers* 2020;105:1–16. 10.1007/s13225-020-00466-2

[129] Zhu K, McCormack ML, Lankau RA, et al. Association of ectomycorrhizal trees with high C soils. *J Ecol* 2018;106:524–35. 10.1111/1365-2745.12918

[130] Payne RJ, Dise NB, Field CD, et al. Nitrogen deposition and plant biodiversity. *Front Ecol Environ* 2017;15:431–6. 10.1002/fee.1528

[131] Schmitz A, Sanders TG, Bolte A, et al. Responses of forest ecosystems to decreasing nitrogen deposition. *Environ Pollut* 2019;244:980–94. 10.1016/j.envpol.2018.09.101 30469293

[132] Phillips RP, Brzostek E, Midgley MG. Mycorrhizal-associated nutrient economy. *New Phytol* 2013;199:41–51. 10.1111/nph.12221 23713553

[133] Klink S, Keller AB, Wild AJ, et al. Fungal residues contribute to mineral-associated organic matter pools. *Soil Biol Biochem* 2022;168:108634. 10.1016/j.soilbio.2022.108634

[134] Martin FM, van der Heijden MGA. The mycorrhizal symbiosis: research frontiers. *New Phytol* 2024;242:1486–506. 10.1111/nph.19541 38297461

[135] Fernandez CW, See CR, Kennedy PG. Decelerated carbon cycling by ectomycorrhizal fungi. *New Phytol* 2020;226:569–82. 10.1111/nph.16269 31622518

[136] Berrios L, Yeam J, Holm L, et al. Positive interactions between mycorrhizal fungi and bacteria benefit plant growth. *Curr Biol* 2023;33:2878–2887.e4. 10.1016/j.cub.2023.06.010 37369208

[137] Lehr NA, Schrey SD, Bauer R, et al. Suppression of plant defence response by a mycorrhiza helper bacterium. *New Phytol* 2007;174:892–903. 10.1111/j.1469-8137.2007.02021.x 17504470

[138] Frey-Klett P, Garbaye J, Tarkka M. The mycorrhiza helper bacteria revisited. *New Phytol* 2007;176:22–36. 10.1111/j.1469-8137.2007.02191.x 17803639

[139] Wang G, Jin Z, George TS, et al. Arbuscular mycorrhizal fungi enhance phosphorus uptake. *New Phytol* 2023;238:2578–93. 10.1111/nph.18772 36694293

[140] Brzostek ER, Rebel KT, Smit KR, et al. Integrating mycorrhizas into global scale models: A journey toward relevance in the earth’s climate system. In: Mycorrhizal Mediation of Soil. Amsterdam: Elsevier, 2017, 479–99. 10.1016/B978-0-12-804312-7.00026-7

[141] Deckmyn G, Flores O, Mayer M, et al. KEYLINK: towards a more integrative soil representation for inclusion in ecosystem scale models. I. review and model concept. *PeerJ* 2020;8:e9750. 10.7717/peerj.9750 32974092 PMC7486829

[142] Huang W, van Bodegom PM, Viskari T, et al. Implementation of mycorrhizal mechanisms into a soil carbon model. *Biogeosciences* 2022;19:1469–90. 10.5194/bg-19-1469-2022

[143] Thurner MA, Caldararu S, Engel J, et al. Modelled forest ecosystem carbon–nitrogen dynamics with integrated mycorrhizal processes under elevated CO_2_. *Biogeosci Discuss* 2024;21:1391–410. 10.5194/bg-21-1391-2024

[144] See CR, Keller AB, Hobbie SE, et al. Hyphae move matter and microbes. *Glob Chang Biol* 2022;28:2527–40. 10.1111/gcb.16073 34989058

[145] Shahzad T, Chenu C, Genet P, et al. Contribution of exudates, arbuscular mycorrhizal fungi and litter depositions to the rhizosphere priming effect induced by grassland species. *Soil Biol Biochem* 2015;80:146–55. 10.1016/j.soilbio.2014.09.023

[146] Sırcan AK, Streck T, Schnepf A, et al. Trait-based modeling of microbial interactions and carbon turnover in the rhizosphere. *Soil Biol Biochem* 2025;202:109698. 10.1016/j.soilbio.2024.109698

[147] Fry EL, De Long JR, Álvarez Garrido L, et al. Using plant, microbe, and soil fauna traits to improve the predictive power of biogeochemical models. *Methods Ecol Evol* 2019;10:146–57. 10.1111/2041-210X.13092

[148] Deckmyn G, Verbeeck H, De Beeck MO, et al. ANAFORE: a stand-scale process-based forest model that includes wood tissue development and labile carbon storage in trees. *Ecol Model* 2008;215:345–68. 10.1016/j.ecolmodel.2008.04.007

[149] Flores O, Deckmyn G, Curiel YJ. KEYLINK: towards a more integrative soil representation for inclusion in ecosystem scale models—II: model description, implementation and testing. *PeerJ* 2021;9:e10707. 10.7717/peerj.10707 33520468 PMC7812927

[150] Guenet B, Flores O, Curiel Yuste J . Deliverable D2.10 ORCHIDEE-Keylink, Holistic management practices, modelling and monitoring for European forest soils (HoliSoils). 2024. https://holisoils.eu/deliverables/. Accessed 8 May 2026.

[151] Mueller GM, Cunha KM, May TW, et al. *Global fungal red list assessments Diversity* 2022;14:736. 10.3390/d14090736

[152] Arnolds E . The fate of hydnoid fungi in the Netherlands and Northwestern Europe. *Fungal Ecol* 2010;3:81–8. 10.1016/j.funeco.2009.05.005

[153] Dahlberg A, Genney DR, Heilmann-Clausen J. Developing a comprehensive strategy for fungal conservation in Europe: current status and future needs. *Fungal Ecol* 2010;3:50–64. 10.1016/j.funeco.2009.10.004

[154] Heilmann-Clausen J, Barron ES, Boddy L, et al. A fungal perspective on conservation biology. *Conserv Biol* 2015;29:61–8. 10.1111/cobi.12388 25185751

[155] Fisher JB, Sweeney S, Brzostek ER, et al. Tree-mycorrhizal associations detected remotely from canopy spectral properties. *Glob Chang Biol* 2016;22:2596–607. 10.1111/gcb.13264 27282323

[156] Kothari S, Beauchamp-Rioux R, Blanchard F, et al. Predicting leaf traits across functional groups using reflectance spectroscopy. *New Phytol* 2023;238:549–66. 10.1111/nph.18713 36746189

[157] Moreno-Martínez Á, Camps-Valls G, Kattge J, et al. Global maps of leaf traits using remote sensing. *Remote Sens Environ* 2018;218:69–88. 10.1016/j.rse.2018.09.006

